# A new nail with a locking blade for complex proximal humeral fractures

**DOI:** 10.1007/s00590-016-1817-4

**Published:** 2016-07-26

**Authors:** F. R. Hashmi, Edgar Mayr

**Affiliations:** 1 Warwick Hospital, Lakin Road, Warwickshire, CV34 5BW UK; 2 Department of Trauma, Hand and Reconstructive Surgery, Klinikum Augsburg, Stenglinstr.2, 86156 Augsburg, Germany

**Keywords:** Proximal humeral fractures, Locked blade, Functional outcome, Insufficient primary stability, Calcar, Rotator cuff, Minimal soft tissue damage, Triangular fixation, Outcome measures

## Abstract

**Introduction:**

The objective of this study was to assess the clinical outcome of displaced proximal humerus fracture treated with a new locking blade nail.

**Materials and methods:**

This prospective study included a series of 92 patients with acute fracture of the proximal humerus treated in one hospital level I trauma centre with locking blade nail between December 2010 and December 2013. According to the Neer classification, all fractures were two- to four-part fractures. Age adopted Constant score, DASH and visual analogue scores were used as outcome measures.

**Results:**

A total of 92 patients were enrolled in the study. However, 29 patients were excluded due to loss to follow-up and death. Ultimately, 63 patients were available for final follow-up and data analysis. The mean duration of follow-up was 22 months (range 16–48 months). On average at 1 year, all fractures had united. The mean weighted Constant score was 84.2 % and the median disabilities of the arm, shoulder and hand (DASH) score was 26, the range of elevation was 115 and range of abduction was 97. The head shaft angle was 130, and pain visual analogue was 1.6. We found that 5 of the 63 patients (8 %) demonstrated complications. Two patients (3 %) displayed secondary displacement and require device removal. Two patients (3 %) had impingement due to prominent metal work, and one patient had a superficial wound infection which was treated with a course of antibiotics.

**Conclusion:**

Our study shows excellent results with new locking blade nail for displaced proximal humerus fractures. We think the locking blade nail offers stiff triangular fixation of the head fragment and support of the medial calcar region to prevent secondary varus collapse.

**Level of evidence:**

III.

## Introduction

Proximal humeral fractures are on the rise due to demographic changes and have become the third most common fracture in the elderly population (after fracture of hip and distal radius) [1–3].

It has been predicted that proximal humeral fractures will increase considerably in the next 30 years [4].

The management of proximal humeral fractures is challenging. These fractures pose a problem for choosing the best management modality, especially to preserve bone alignment and joint surface congruity and yet maintain the vascularity of the humeral head. This is especially the case with complex fractures, where there is a significant complication rate; however, they are managed.

Conservative management has a role in un-displaced and minimally displaced fractures but shows consistently inferior results in terms of pain relief and return of functional range of motion [4, 5].

Currently, operative treatment appears to be the preferred method of treatment of these fractures. Surgical management will result in better restoration of anatomy and early physiotherapy will decrease the risk of shoulder stiffness and lead to a better definitive functional outcome [6, 7] which is fundamentally important in keeping the elderly patient independently mobile.

Hertel et al. [8] described predictors of humeral head ischaemia and especially ascribed importance to the integrity of the medial hinge.

Gardner et al. [9] pointed out that achieving mechanical support of the inferomedial region of the proximal humerus is important for maintenance of reduction and eventual healing.

The cancellous bone quality has been already investigated using various techniques in the humeral head based on anatomical landmarks [10–13]. Biomechanical studies and dissection model with 3-D reconstruction had shown the strong bone sites in the humeral head to have good purchase of the screws [14].

The main factors predicting poor outcome are age, fracture pattern especially the medial support, osteoporosis, head-neck shaft angle and co-morbidity [15, 16]. Anatomical reduction and medial support are the only factors that can be influenced.

Recent advances in anatomically designed locking plates and nails have significantly improved the stability of fixation [17–19].

By combining axial and angular stability, locking plate fixation provides increased osseous anchorage and high failure loads [20]. Although locking plate fixation has become a standard treatment with good short-term functional results–complication rates are still unsatisfactory especially the avascular necrosis due to soft-tissue stripping and screw penetration [21].

By comparison, proximal nails have also gained popularity in the management of these fractures due to minimal soft-tissue stripping and ease of use. The nails are load-sharing devices and demonstrate higher stiffness values.

The main objection against nail usage is their insufficient primary stability and loss of medial support and rotator cuff damage at insertion [22].

We present a new locking blade nail with straight design, distally inserted locking blade for medial support and locking screws with mobile washers.

The straight design helps prevent rotator cuff damage through entry point medial to rotator cuff insertion, and the locking blade provides triangular stability to maintain continued medial calcar support and locking screws with washers provide primary stability and fixation of greater and lesser humeral tuberosities.

## Materials and methods

This prospective study included a series of 92 patients with acute fractures of the proximal humerus treated in one hospital level I trauma centre with locking blade nail (Marquardt Medizintechnik Europe) between December 2010 and December 2013. Approval from the local ethics committee and the institutional review board was granted. All patients gave written informed consent prior to study participation.

Patients presenting with proximal humerus fracture at our level I trauma centre, for which a collective decision of surgical treatment was taken, were included in the study. The following exclusion criteria were applied: age <50 year old, unable to give informed consent, open fractures and proximal humerus fractures associated with head splitting.

According to the Neer classification, all fractures were two- to four-part fractures with >45* of angulation or >1 cm of displacement between major fracture fragments.

Preoperative plain radiograph (anteroposterior and later view) and CT were used to detail the pattern of the fracture, with 3-D reconstruction to estimate the extent of comminution, articular involvement and amount of tuberosity displacement.

### Characteristic of the nail

The nail (made of titanium alloy) is straight with a choice of four proximal cross-locking screws and one distal locking screw. The proximal screws are two for the middle region of the humeral head and one each anterior and posterior for lesser and greater tuberosity fixation. The two middle screws also lock the blade proximally.

The single locking blade for calcar support has the option of a small or large blade. The blade locks into position in the nail distally and middle screws proximally. Along with two middle screws, the locking blade provides high triangular stability to the medial calcar and metaphyseal region of the humeral head.

The mobile washers in the proximal screws provide optional anchorage for suture fixation.

**Fig. 1-2:**
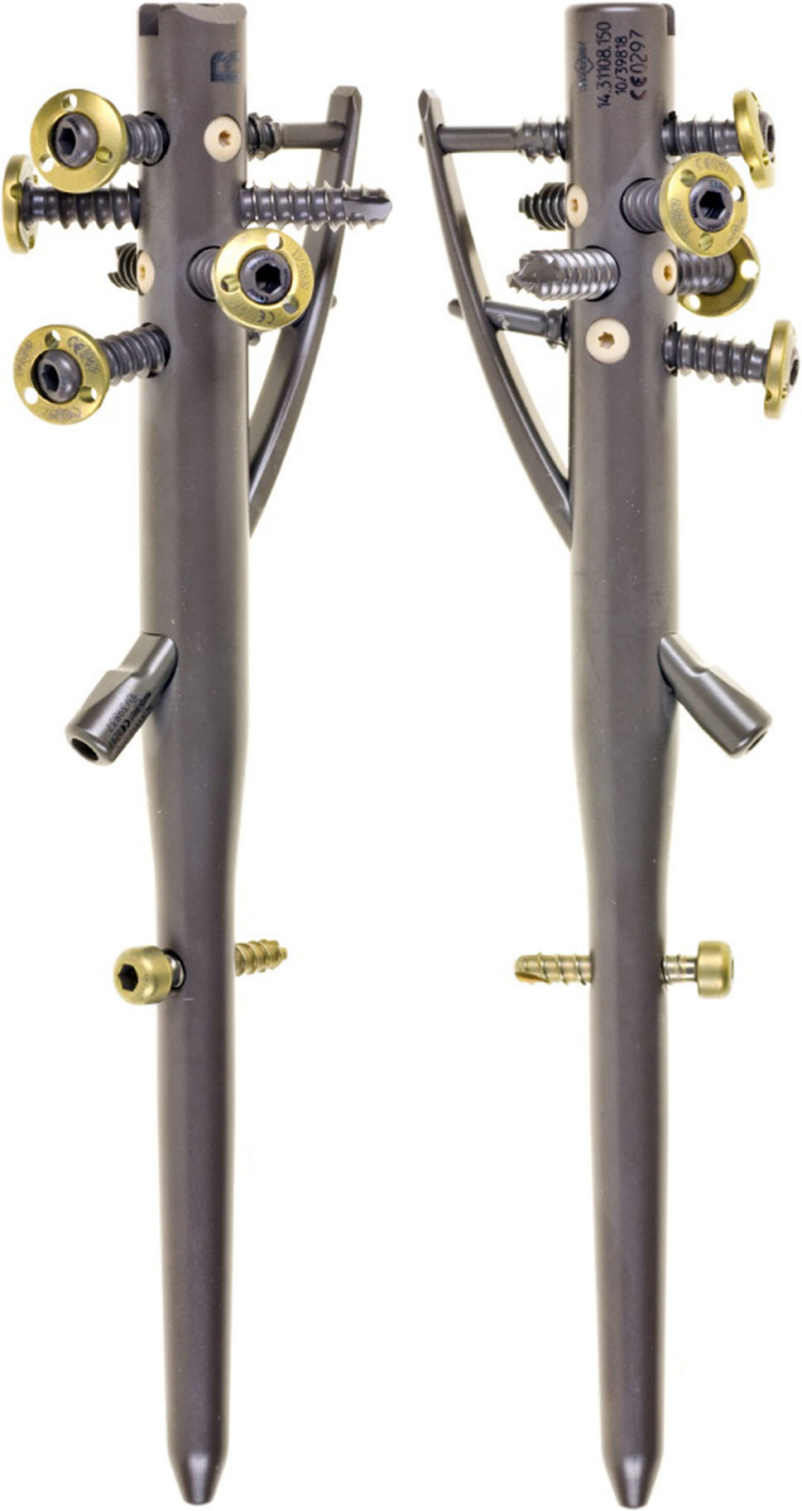
Fully assembled LBN (locking blade nail) with blade and locking screws

The standard proximal humeral nail is 150 mm long and acts as a central pillar against secondary collapse. The straight shape of the nail helps protect the rotator cuff (Fig. 1-2) with entry point medial to the insertion of rotator cuff.

### Surgery and Assessment methods

**Fig. 3-4:**
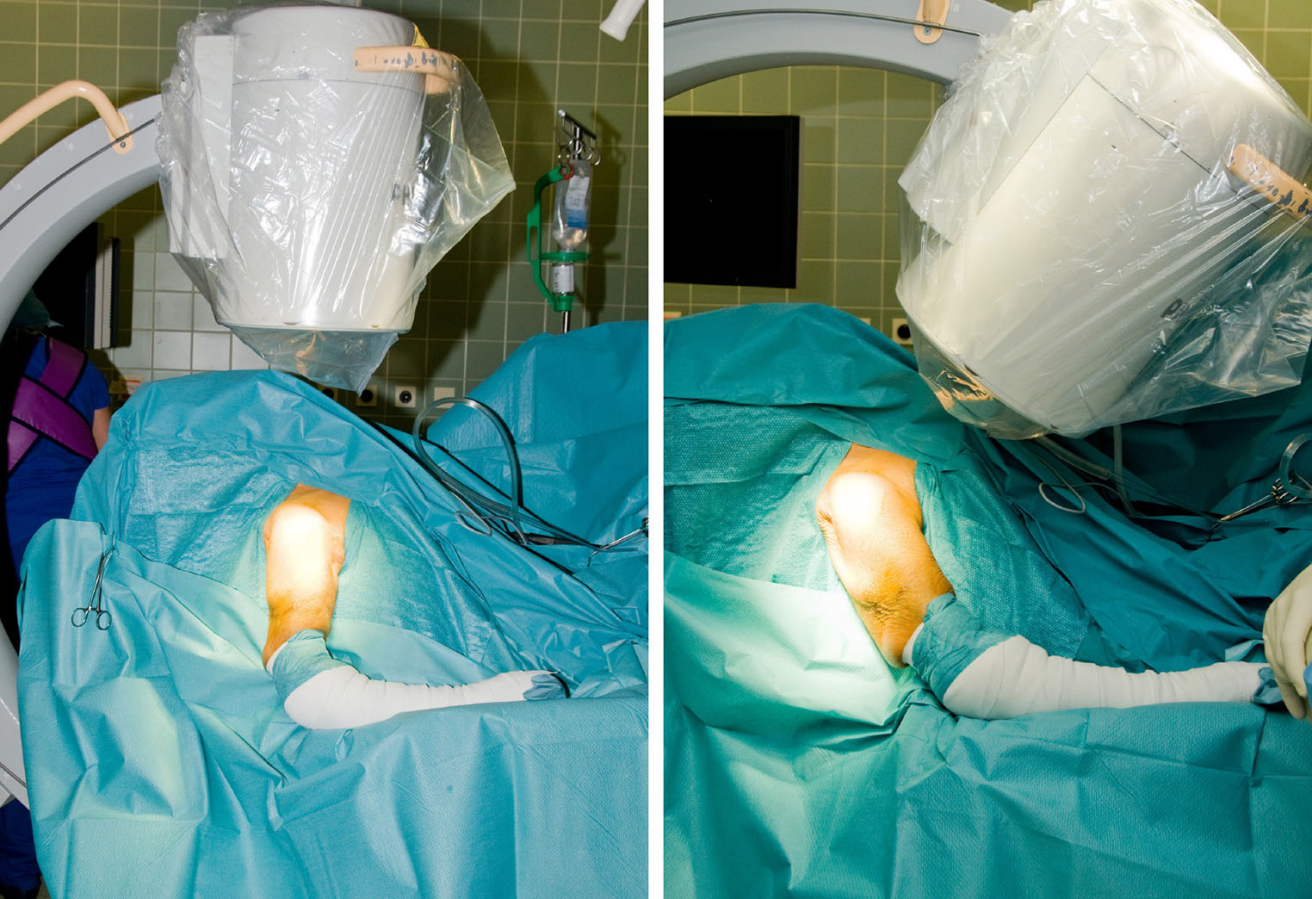
Position of X-ray tube for screening during LBN insertion

All patients had their surgery within 36 h of their injuries under general anaesthesia; patients were placed in a beach chair position with the forearm supported by an arm rest. It was important that the shoulder protruded out of the table and the arm was longitudinally straight with elbow free and mobile. The image intensifier was positioned posterior to shoulder right over the shoulder for lateral view, and C-arm was rotated over the shoulder for anteroposterior view (Fig. 3-4).

**Fig. 5-6:**
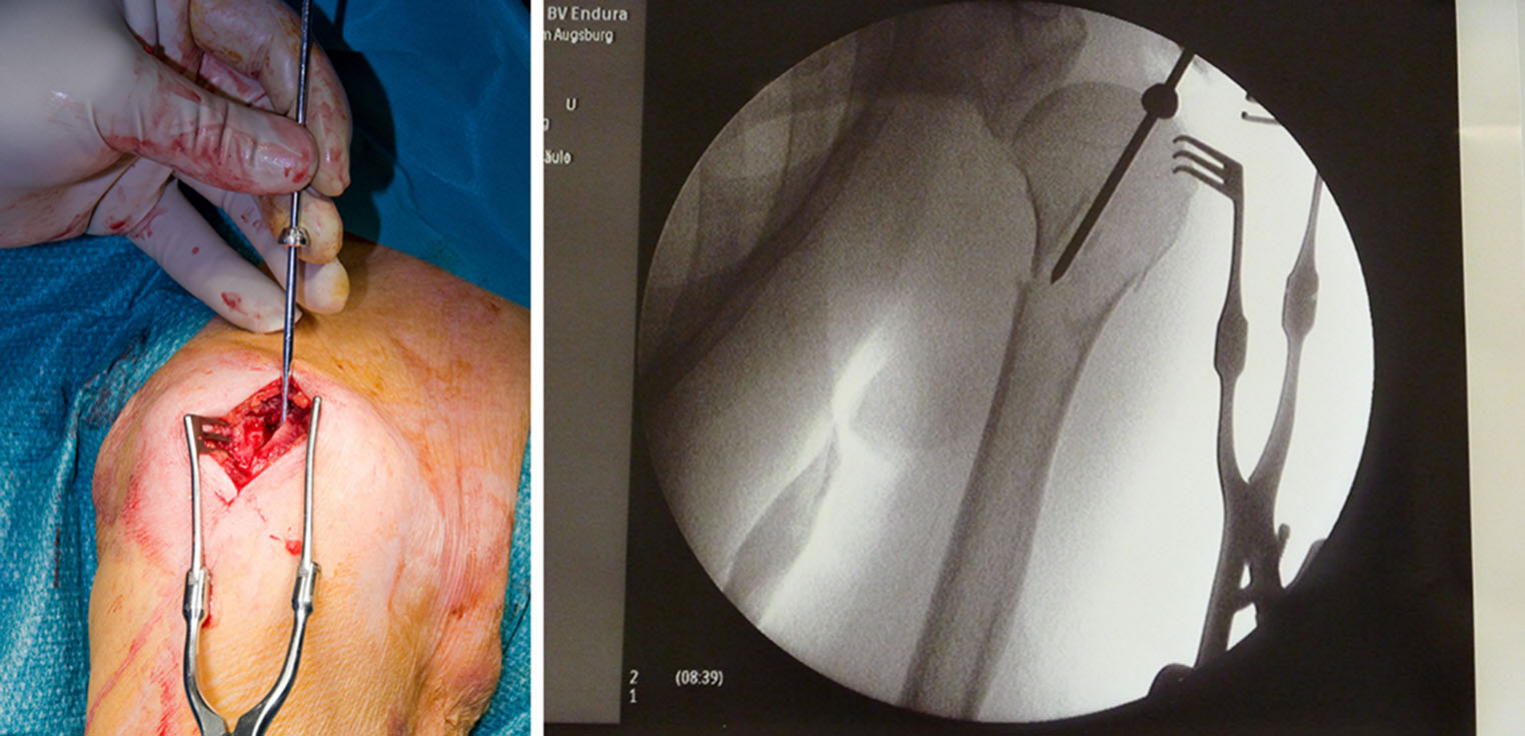
Rotator cuff split and guide wire position and entry into proximal humerus

**Fig. 7:**
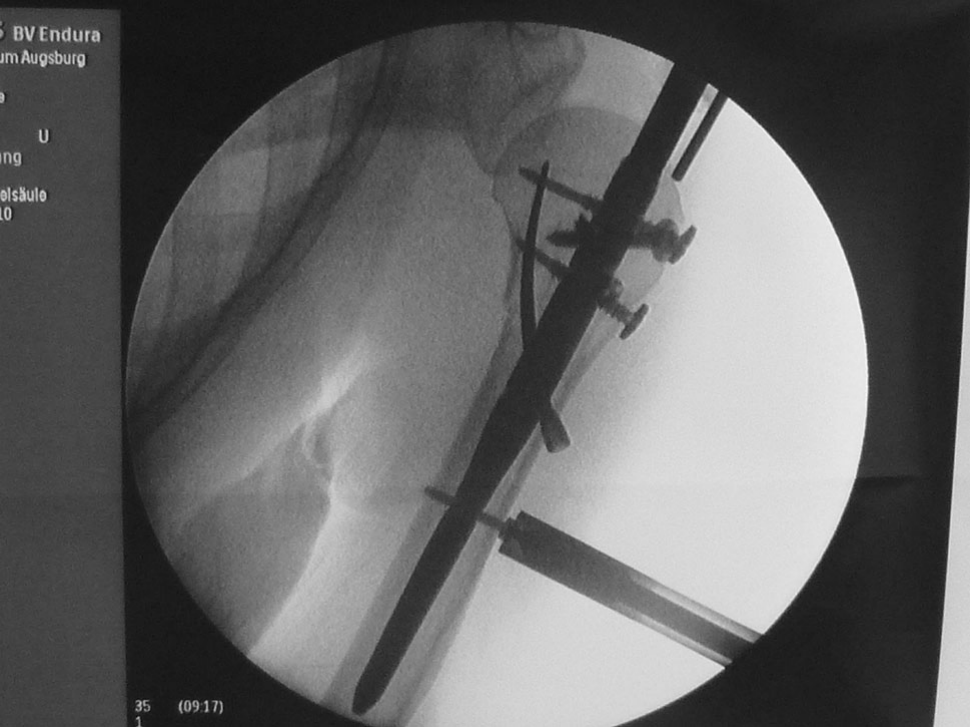
Intraoperative LBN insertion and fixation proximally and distally

After the routine, anterolaterally directed 3–4 cm skin incision from the tip of the acromion, the deltoid muscle was split in line with skin incision and rotator cuff was visualised. After reduction of the fracture under image intensifier, nail entry point was selected medial to rotator cuff insertion. Rotator cuff was longitudinally split. After confirmation of reduction, a guide wire was passed under image intensifier (Fig. 5-6). After reaming the entry point, the nail was inserted and fixed proximally and distally (Fig. 7).

All patients started physiotherapy 24 h after surgery, and active mobilisation began at 6 weeks.

**Fig. 8-10:**
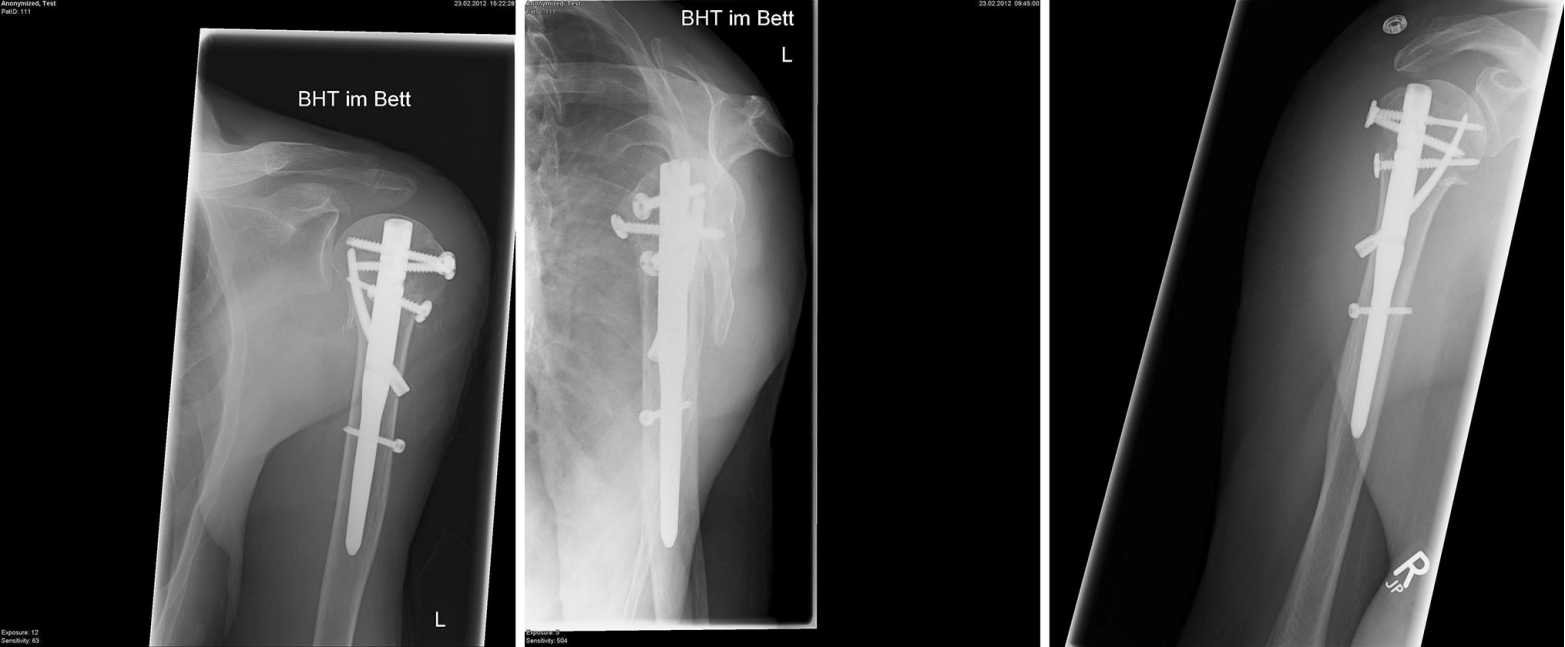
Post-operative radiograph showing LBN in position

Follow-up was conducted at 2, 6 weeks, 3, 6 months, 1 and 2 years post-surgery. At each follow-up visit, patients were seen by the attending surgeon along with the research coordinator. The primary outcome measure was the clinical Constant score adjusted for age and gender. Secondary outcome measures included radiological analysis of the fracture reduction (Fig. 8-10) especially head shaft angle and the occurrence of adverse events, such as infection, osteonecrosis, construct failure, secondary displacement, algodystrophy and reoperation. Disabilities of the arm, shoulder and hand (DASH) and visual analogue (VAS) scores were used to measure quality of life.

Descriptive statistics were computed. Qualitative variables were described as frequency and percentage and quantitative variables as mean minimum and maximum.

## Results

A total of 92 patients were eligible and enrolled in the study initially. However, 29 patients were excluded due to the following: lost to follow-up (*n* = 17) or died (*n* = 12) prior to completion of study. Ultimately, 63 patients were available for final follow-up and data analysis. There were nine two-part, 45 three-part and nine were four-part proximal humeral fractures.

Thus, the final study population consisted of 50 women and 13 men with a mean age of 67.5 years old (range 53–90 years) (Table 1).

**Table 1:** Patient’s gender and types of fractures

Type of fracture	Two-part fracture	Three-part fracture	Four-part fracture
No. of patients	9	45	9
Men	3	8	2
Women	5	33	12

### Clinical scores

The mean duration of follow-up was 22 months (range 16–48 months). On average at 1 year, all fractures had united. The mean Constant score was 62 (48–76). The weighted Constant score was 84.2 % (65–102) and the median disabilities of the arm, shoulder and hand (DASH) score was 26 ± 20, the range of elevation was 115 ± 20 and range of abduction was 97 ± 21. The head shaft angle was 130 ± 7, and visual analogue score was 1.6 ± 1 (Table 2).

**Table 2:** Outcome measures used for assessments

Outcome measures	3 months	6 months	Final follow-up
Constant score	49.0 ± 14	60.7 ± 16.7	62 ± 14
Weighted Constant score	49.9 ± 19.4	60.3 ± 22	84.2 ± 18.3
DASH score		28.4 ± 23.0	26.0 ± 20.5
ROM abduction	84* ± 28	94 ± 20	97 ± 21
ROM elevation	94* ± 44	110 ± 21	115 ± 18
Head shaft angle	130* ± 6.7	130* ± 7	130 ± 7
VAS	2.8 ± 1.8	1.6 ± 1	1.0 ± 1

### Complications

We found that 5 of the 63 patients (8 %) demonstrated post-operative complications. Two patients (3 %) displayed secondary displacement and belong to four-part fracture pattern group. Both of these cases were associated with osteonecrosis of the humeral heads and required device removal and total shoulder arthroplasty. Two patients (3 %) had impingement due to prominent metal work belonging to two-part fracture pattern group. Both of these cases needed earlier removal of metal work; one patient had wound infection which was resolved with a course of antibiotics.

## Discussion

In the present study, we assessed the clinical as we as radiological results and complications associated with a consecutive series of proximal humeral fractures treated with a new locking blade nail. We found that this technique showed good clinical results with low complications. The fundamental principles behind using the locking blade nail were that (a) humeral medial calcar alignment and support is necessary for fracture healing [8]; (b) stability of humeral head is required in anatomical position while healing is taking place [9]; and (c) minimal rotator cuff and soft tissues damage are important for shoulder function and preventing avascular necrosis of humeral head and screws cut-out [10].

Based on these principles, antegrade nailing of the proximal humerus with the locking blade nail (LBN) provides strong calcar support by the distally inserted blade which not only stabilises and supports the calcar but also the medial head by keeping it in anatomically reduced position. The blade with two proximal locking screws prevents head rotation and varus collapse which is a common deformity after final fracture healing. It provides the triangular support to the metaphyseal region of the humeral head and counteracts the supero-medially directed forces of rotator cuff. The force to counteract rotator cuff action is an essential component of keeping the fracture in anatomical reduced position, while healing is taking place.

We used the weighted Constant score for clinical evaluation of results in our study. This scoring system is more representative of functional outcome as it takes away gender differences in muscular strength in the elderly population. In this regard, we observed a mean weighted Constant score of 84 %, which is a satisfactory despite the existence of an 8 % complication rate. Thus, the clinical results from our patient population appear to be satisfactory in comparison with the current data on proximal humeral nailing [23, 24].

Finally, this study also has a few limitations. In particular, a large proportion of patients were lost to follow-up, which may have biased our results. However, since this study was conducted in injured elderly patients, it is difficult to avoid incomplete follow-up within this fragile population. The small sample size may not have been sufficient for accurately assessing the efficacy of this technique for each of the fracture types.

## Conclusion

Our study shows excellent results with new locking blade nail for complex proximal humerus fractures. We think the locking blade nail offers stiff triangular fixation of the head fragment and support of the medial calcar region to prevent secondary varus collapse.
